# Anlotinib for Recurrent or Metastatic Primary Malignant Bone Tumor: A Multicenter, Single-Arm Trial

**DOI:** 10.3389/fonc.2022.811687

**Published:** 2022-05-26

**Authors:** Lina Tang, Xiaohui Niu, Zhen Wang, Qiqing Cai, Chongqi Tu, Zhengfu Fan, Yang Yao

**Affiliations:** ^1^ Shanghai 6th People’s Hospital, Shanghai Jiao Tong University, Shanghai, China; ^2^ Beijing Jishuitan Hospital, Peking University, Beijing, China; ^3^ Department of Orthopaedics, The First Affiliated Hospital, The Air Force Medical University, Xi’an, China; ^4^ Henan Cancer Hospital, Zhengzhou University, Zhengzhou, China; ^5^ Department of Orthopaedics, West China Hospital, Sichuan University, Chengdu, China; ^6^ Beijing Cancer Hospital, Peking University, Beijing, China

**Keywords:** osteosarcoma, chondrosarcoma, multitarget tyrosine kinase inhibitor, angiogenesis, anlotinib, progress-free survival

## Abstract

**Objective:**

Anlotinib, a novel multitarget kinase inhibitor of VEGFR, FGFR, PDGFR and c-Kit, has proven to be effective and safe for refractory soft tissue sarcoma patients, but has not been examined in recurrent or metastatic primary malignant bone tumors in a clinical trial setting.

**Methods:**

This is a multicenter single-arm trial. Patients with pathologically proven recurrent or metastatic primary malignant bone tumors were eligible. Anlotinib was administered orally at 12 mg per day. Each cycle consisted of 2 weeks of treatment followed by 1-week off-treatment. The primary endpoint was progression-free survival (PFS), as assessed in the intention-to-treat (ITT) population. Secondary endpoints included objective response rate (ORR), disease control rate (DCR) and overall survival (OS). Adverse events (AEs) were assessed per NCI CTCAE version 4.03.

**Results:**

A total of 42 patients were enrolled. Median PFS was 5.3 months (95% CI 3.5-8.4 months) in the overall analysis, 4.8 months (95%CI 3.5-7.1 months) in osteosarcoma patients and 2.8 months [95%CI 1.3 months to not reached (NR)] in chondrosarcoma patients. The median OS was 11.4 months (95% CI 10.1 months to NR) in the overall analysis, not reached (95% CI, NR, NR) in osteosarcoma patients and 11.4 months (95% CI 1.8 to 21.1 months) in chondrosarcoma patients. The ORR was 9.52% and DCR was 78.57%. Grade 3 or above AEs occurred in 54.76% of the patients, and included hypertension (19.05%), hypertriglyceridemia (9.52%) and pustulosis palmaris et plantaris (7.14%). No treatment-related death was reported.

**Conclusion:**

Anlotinib demonstrated promising antitumor activities in recurrent or metastatic primary malignant bone tumors with manageable AEs.

## Introduction

Osteosarcoma, chondrosarcoma, Ewing’s sarcoma are common subtypes of malignant bone tumors. Osteosarcoma most commonly affects children, adolescents, and young adults, accounting for 1–2% of all adult cancers (1). Primary therapy for osteosarcoma typically consists of surgical resection and neoadjuvant and adjuvant chemotherapy with doxorubicin, cisplatin, ifosfamide and methotrexate. These treatments have dramatically improved the prognosis of patients with localized osteosarcoma, with a long-term survival rate of 65% to 70% (2). In the absence of targeted therapy, salvage treatment options such as with ifosfamide or gemcitabine plus docetaxel have yielded a rather dismal clinical outcome in osteosarcoma patients who are refractory to chemotherapy or who experience relapse with metastatic disease, with a 4-month progression-free survival (PFS) rate of as low as 12% (3, 4). Genetically, osteosarcoma is characterized by few recurrent genetic alterations and carries mainly somatic copy number alterations (5). The lack of targetable recurrent gene mutations renders molecularly targeted therapy challenging and so far largely disappointing (6). In addition, immune checkpoint inhibitors (ICIs) including those targeting programmed cell death protein-1 (PD-1)/PD-ligand 1 (PD-L1) have demonstrated scant activity in osteosarcoma (7).

Ewing sarcoma, an aggressive sarcoma of bone and soft tissue, may occur at any age with a peak incidence in adolescents and young adults. Primary therapy for Ewing sarcoma mainly consists of surgery, intensive neoadjuvant and adjuvant chemotherapies and/or radiotherapy, with a 5-year overall survival (OS) of 65% to 75% for patients with localized Ewing sarcoma. Despite extensive surgical resection, aggressive chemotherapy, and radiotherapy, the 5-year OS of recurrent or metastatic Ewing sarcoma is less than 30% (8). Chondrosarcoma is inherently resistant to both chemotherapy and radiotherapy and the 5-year survival stands at merely 5% after recurrence (9). Therefore, novel effective therapeutic agents or regimens are urgently needed.

Accumulating evidence suggests that multitarget tyrosine kinase inhibitors (TKIs) have antitumor activities in osteosarcoma. TKIs have been recommended by the National Comprehensive Cancer Network (NCCN) guidelines as second-line therapy for advanced osteosarcoma that progresses after chemotherapy (10). In phase II trials, sorafenib achieved a 4-month PFS rate of 46% in advanced osteosarcoma patients who had failed standard therapy while apatinib achieved a 4-month PFS rate of 56.76% in advanced osteosarcoma patients who had failed chemotherapy (11, 12). In SARC024, a randomized, placebo-controlled phase II trial in patients with progressive metastatic osteosarcoma, regorafenib prolonged median PFS from 1.7 months [95% confidence interval (95% CI): 12-1.8] to 3.6 months (95%CI: 2.0-7.6). The hazard ratio (HR) was 0.42 (95%CI: 0.21-0.85) (13). Though these studies suggest that multitarget TKIs could offer a promising treatment for recurrent metastatic primary malignant bone tumors, improvement in objective response rate (ORR), disease control rate (DCR) or PFS is modest and there is an urgent need for more effective, novel multikinase inhibitors (MKIs) for advanced osteosarcoma.

Anlotinib is a novel MKI that exerts its antitumor activities by inhibiting angiogenesis and suppressing tumor cell proliferation *via* blocking vascular endothelial growth factor receptors (VEGFR), fibroblast growth factor receptor (FGFR), platelet-derived growth factor receptor (PDGFR), and c-Kit (14). Anlotinib has demonstrated stronger *in vitro* and *in vivo* anti-angiogenesis activities than other antiangiogenic agents such as lenvatinib and sorafenib, with an IC_50_ of 0.2, 0.7 and 14.8 nmol/L for VEGFR-2, VEGFR-3 and KIT, respectively, *vs.* 4, 5.2 and 100 nmol/L for lenvatinib and 90, 20 and 68 nmol/L KIT for sorafenib (15–19). The efficacy and safety of anlotinib have been established in several tumor types including NSCLC and advanced soft tissue sarcoma (20, 21). A phase II trial of 166 refractory soft tissue sarcoma patients demonstrated that anlotinib achieved an ORR of 13% (95%CI 7.6%-18%), a median PFS of 5.6 months and OS of 12 months with acceptable toxicities (22).

The efficacy and safety of anlotinib have not been examined in recurrent metastatic primary malignant bone tumors in a clinical trial setting. Given the promising antitumor activities of anlotinib in soft tissue sarcoma and other tumor types, we hypothesized that anlotinib could be effective for recurrent or metastatic primary malignant bone tumors.

## Patients and Methods

### The Study Design and Population

This multicenter, single-arm trial enrolled patients (14 ~70 years of age) with pathologically confirmed primary malignant bone tumors including osteosarcoma, chondrosarcoma, undifferentiated pleomorphic sarcoma of bone/malignant fibrous histiocytoma (MFH) of the bone, giant cell tumor of the bone and Ewing sarcoma/primitive neuroectodermal tumor (PNET). Patients were diagnosed as having recurrent or metastatic disease after surgery and adjuvant therapy. Other inclusion criteria were: at least one measurable lesion according to RECIST 1.1; Eastern Cooperative Oncology Group (ECOG) performance status score of 0 or 1 (except for amputees); treatment failure after anthracyclines-based chemotherapy, or patients could not tolerate chemotherapy (patients with chondrosarcoma or giant cell tumor of the bone were not required to have received first line chemotherapy). We excluded patients who had participated in clinical trials of anlotinib therapy or who had received VEGF inhibitors. Patients with symptomatic brain metastasis or brain metastasis controlled for less than 2 months were also ineligible. Additional eligibility criteria are provided in **Supplementary Methods**.

The trial was conducted in accordance with the provisions of the Declaration of Helsinki and the International Conference on Harmonisation guidelines for Good Clinical Practice and approved by the institutional review board of Shanghai Sixth People’s Hospital (China). Written informed consent was obtained before enrollment from patients and parents of pediatric patients or their legal surrogates or guardians. The trial is registered with http://www.clinicaltrials.gov (NCT03527888). The study protocol adhered to the SPIRIT statement and the reporting of the study adhered to the CONSORT statement (23, 24).

### Treatment

Anlotinib (12 mg/d) was administered orally. Each cycle consisted of 2 weeks of treatment followed by 1 week off treatment (25). Treatment was continued until disease progression per RECIST 1.1, intolerable toxicity or at the discretion of the investigator. Dose adjustment of anlotinib was allowed at the discretion of the investigator upon drug-related adverse events (AEs) per National Cancer Institute Common Terminology Criteria for Adverse Events version 4.03 (NCI CTC AE 4.03). Two dose reductions were allowed: from 12 mg/d to 10 mg/d and from 10 mg/d to 8 mg/d.

Best supportive care was provided. Patients were allowed to receive bisphosphonates for bone metastasis and, psychological therapy, or other symptomatic treatment. In addition, palliative radiotherapy for the non-target metastatic lesions at bone was allowed to control bone metastasis-associated pain with irradiation field confined to less than 5% of the bone marrow (26). Drugs that could interfere with the study medications were not allowed.

### Tumor Response Assessment

Responses were evaluated by an independent review committee according to RECIST v1.1 using computed tomography (CT) or magnetic resonance imaging (MRI) within 2 weeks of study entry, at 6 weeks post treatment and once every two cycles thereafter. CR, PR, and SD had to be confirmed with a repeat scan after at least 6 weeks.

### Outcomes

The primary endpoint was PFS, defined as the time from the date of administration of the first dose of anlotinib to the date of disease progression or death of all causes. Secondary endpoints included ORR, which was the proportion of all evaluable patients who achieved CR or PR per RECIST v1.1, DCR, which was the proportion of patients who achieved CR, PR, or SD lasting for at least 4 weeks, and OS, which was calculated from the date of administration of the first dose of anlotinib to the date of death from any cause.

### Safety Evaluation

Data for AEs were recorded from the first date of drug administration until one month after treatment termination, and graded according to NCI CTCAE version 4.03. Safety assessments were based mainly on the occurrence, frequency, severity of AEs and the correlation with study drug.

### Statistical Analysis

The median PFS of patients with relapsed osteosarcoma who had received chemotherapy was between 2 to 3 months (3, 27, 28). We hypothesized that anlotinib treatment can prolong the median PFS from 2.5 months to 4.2 months. A total of 37 patients were required to detect a 41% decrease in HR for progression based on 80.2% power and one-sided test at a significance level of 0.025. Assuming a dropout rate of 10%, we planned to enroll 42 patients.

All statistical analysis was undertaken using SAS version 9.2 (SAS Institute Inc., Carry, North Carolina, United States). Statistical analyses were prespecified and followed the intention-to-treat (ITT) principle.

Quantitative variables were expressed in mean and standard deviation or median (range or interquartile range, IQR). Changes from baseline within a group were analyzed by Student’s *t* test for paired data. Categorical variables were described in frequency and percentage and changes from baseline within a group were analyzed by chi-square (χ2) test or Fisher’s exact test. PFS and OS were estimated by the Kaplan-Meier method and expressed in median (95%CI). The safety set included all patients who received at least one dose of the study drug and had safety assessment.

All tests were two-tailed with a level of significance set at α = 0.05.

## Results

### Patients

**Figure 1:**
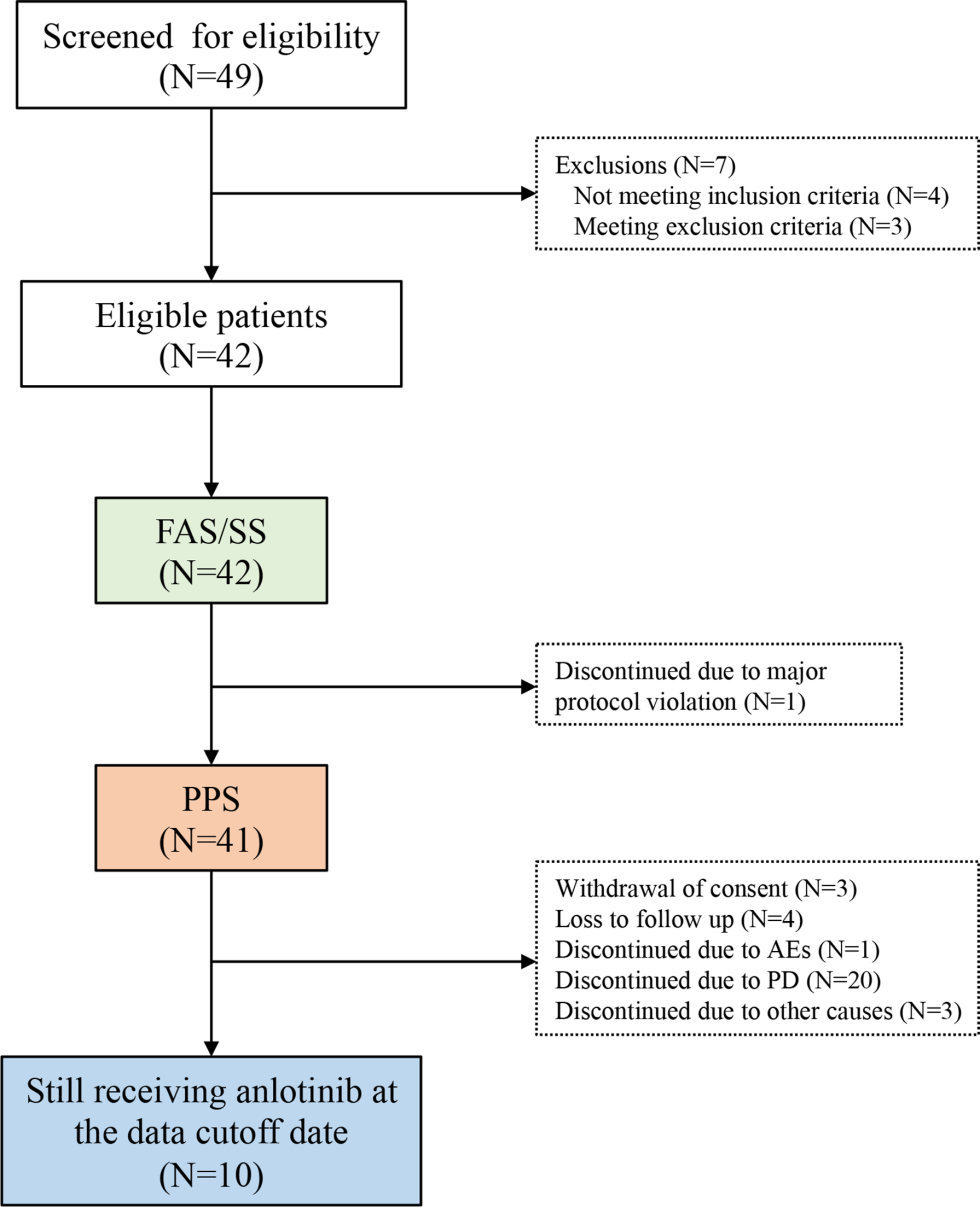
The study flowchart. AE, adverse event; FAS, full analysis set; ITT, intention-to-treat; PPS, per-protocol set; SS, safety set.

Forty-nine patients were screened for eligibility between September 1, 2018 and April 30, 2019; 42 patients were enrolled (**Figure 1**). Their median age was 28.0 years (IQR: 18.0, 36.0) and 59.5% of them were male. Most patients were diagnosed with osteosarcoma (n=29; 69.05%) or chondrosarcoma (n=9; 21.43%). Furthermore, 25 (59.52%) and 15 (35.71%) patients had stage IVA and IVB disease, respectively, and 39 (92.86%) patients had lung metastasis. All patients received chemotherapy in the adjuvant setting and 7 (16.67%) patients received at least 2 prior lines of chemotherapy. Patient demographic and baseline characteristics are shown in **Table 1**.

**Table 1:** Patient demographic and baseline characteristics.

Variables	N = 42
**Age, years**	
Median (IQR)	28.0 (18.0, 36.0)
≤65	41 (97.62)
**Male gender, n(%)**	25 (59.5)
**ECOG performance status score, n(%)**	
0	3 (7.14)
1	34 (80.95)
2 and above	5 (11.9)
**Pathological subtypes, n(%)**	
Osteosarcoma	29 (69.05)
Chondrosarcoma^	9 (21.43)
Ewing sarcoma/PNET*	3 (7.14)
Malignant fibrous histiocytoma (MFH) of bone	1 (2.38)
**Clinical stage, n(%)**	
IIB	1 (2.38)
III	1 (2.38)
IVA	25 (59.52)
IVB	15 (35.71)
**Previous surgery, n(%)**	
Yes	42 (100)
**Previous radiotherapy, n(%)**	
Yes	6 (14.29)
**Previous targeted therapy, n(%)**	
Yes	2 (4.76)
**Metastatic at diagnose**	39 (92.6)
Lung metastasis	39 (92.6)
Metastasis to other sites	13 (31.0)
**Adjuvant therapy, n(%)**	42 (100)
**First line chemotherapy, n(%)**	39 (92.9)
**Second-line chemotherapy, n(%)**	7 (16.7)
**Third-line Chemotherapy, n(%)**	1(2.4)
**Unclear**	3 (7.14)

*PNET, primitive neuroectodermal tumor.

^Including 5 cases of mesenchymal chondrosarcoma, 2 cases of grade 2 chondrosarcoma, 1 case of grade 3 chondrosarcoma and 1 case of dedifferentiated chondrosarcoma.

### Efficacy Measures

**Figure 2:**
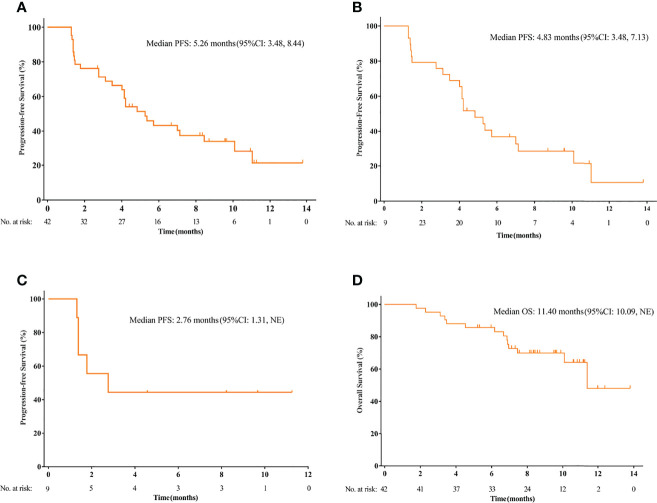
The Kaplan-Meier curve of progression-free survival (PFS) in the intention-to treat (ITT) population **(A)**, and the subsets of osteosarcoma patients **(B)** and chondrosarcoma patients **(C)**. **(D)** The Kaplan-Meier curve of overall survival (OS) in the ITT population.

The median duration of follow up was 9.6 months (95%CI 8.4-10.8). The data cutoff date was January 1, 2020. PFS events occurred in 28 (66.7%) patients. The median PFS was 5.3 months (95%CI 3.5-8.4 months) and the 3-month PFS rate was 71.27% (**Figure 2A**). In patients with osteosarcoma, the median PFS was 4.8 months (95%CI 3.5-7.1 months) and the 3-month PFS rate was 75.86% (**Figure 2B**). In patients with chondrosarcoma, the median PFS was 2.8 months [95%CI 1.3 months to not reached (NR)] and the 3-month PFS rate was 44.44% (**Figure 2C**). Only 3 patients with Ewing sarcoma/PNET were enrolled. The PFS was 8.3, 8.4 and 11.1 months, respectively. OS events occurred in 14 (28.6%) patients. The median OS was 11.4 months (95% CI 10.1 months to NR) (**Figure 2D**). The median OS was not reached for osteosarcoma (95% CI, NR, NR) and was 11.4 months (95% CI 1.8 to 21.1 months) for chondrosarcoma.

**Figure 3:**
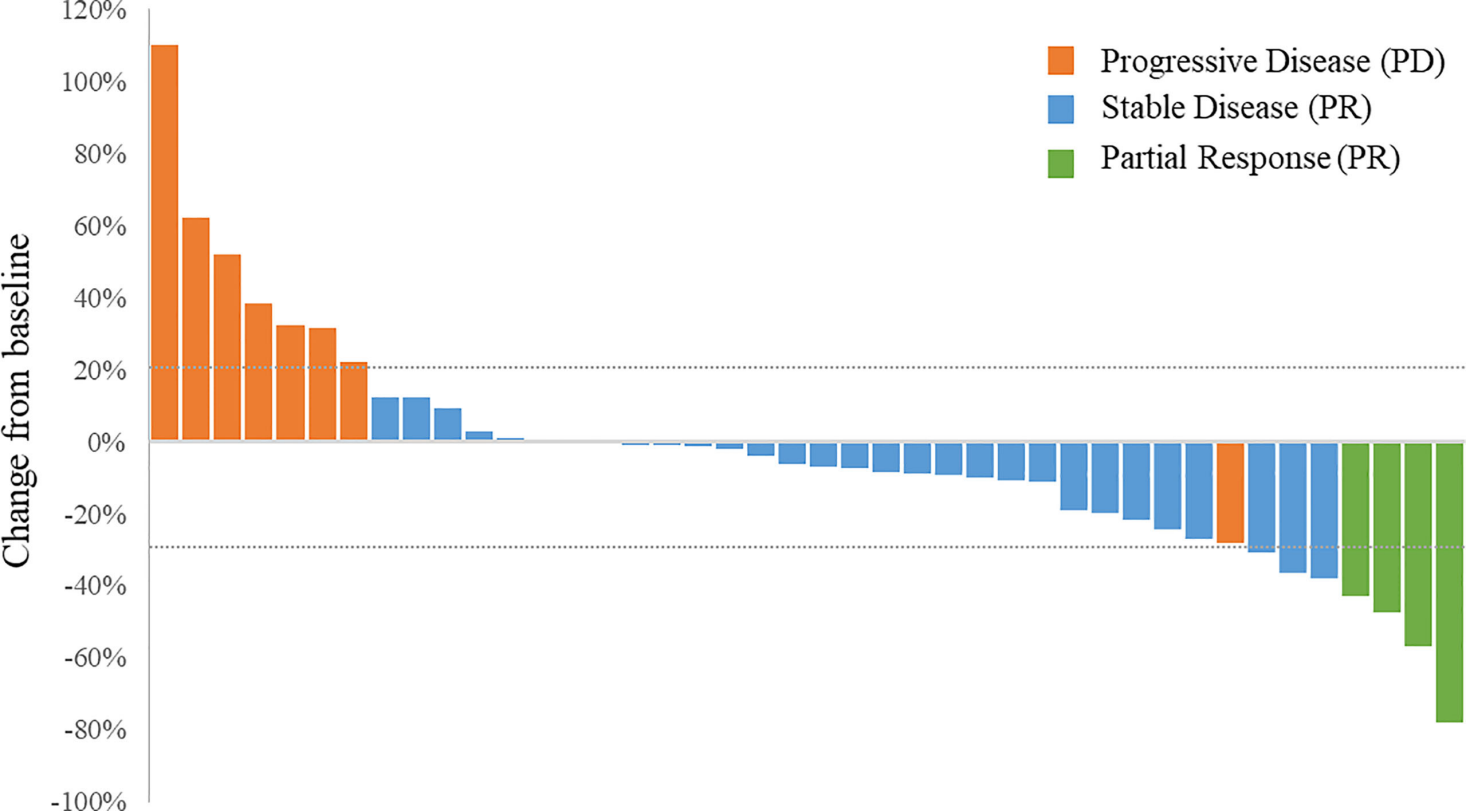
Waterfall plot of the best percentage changes for the sum of target lesion diameters after anlotinib treatment are shown for individual patients with best objective response per RECIST version 1.1 as indicated by the color codes. CR, complete response; PD, progressive disease; PR, partial response; SD, stable disease.

No patient achieved CR. Notably, 4 patients, including 2 osteosarcoma patients achieved PR (**Figure 3**). Tumor response was observed in 4 patients and the ORR was 9.52% for the overall population. Two patients with osteosarcoma achieved PR and the ORR was 6.90%. In addition, 29 patients had SD and the DCR was 78.57% for the whole population. The DCR for patients with osteosarcoma was 75.86%. Among the 3 patients with Ewing sarcoma/PNET, 2 achieved PR and 1 had SD. The ORR was 66.67% and the DCR was 100% (**Table 2**).

**Table 2:** Best overall responses by tumor types.

Bone tumors	N	CRn	PRn	SDn	PDn	ORRn (%)	DCRn (%)
**Osteosarcoma**	**29**	0	2	20	7	2 (6.90)	22 (75.86)
**Chondrosarcoma**	**9**	0	0	7	2	0 (0)	7 (77.78)
**Ewing sarcoma/PNET tumor**	**3**	0	2	1	0	2 (66.67)	3 (100)
**Malignant fibrous histiocytoma of bone**	**1**	0	0	1	0	0 (0)	1 (100)
**Total**	**42**	0	4	29	9	4 (9.52)	33 (78.57)

The levels of response were evaluated by independent radiologic review per RECIST version 1.1.

CR, complete response; DCR, Diseace control rate; ORR, objective response rate; OS, overall survival; PD, progressive disease; PFS, progression-free survival; PNET tumor, Primitive neuroectodermal tumor; PR, partial response; SD, stable disease.

The objective response rate was the percentage of patients who had a best-response rating of CR or PR per RECIST Version 1.1 based on independent radiologic review. The disease-control rate was the proportion of patients who had a best-response rating of CR or PR or SD per RECIST Version 1.1 based on independent radiologic review.

### Safety

The median treatment cycles was 7 (range 2 to 22). Seven (16.67%) patients underwent dose reductions from 12 mg/d to 10 mg/d. One of them was still intolerable to anlotinib at the reduced dose and permanently discontinued treatment eventually due to AE. Dose modifications are detailed in **Supplementary Table 1**. Twenty patients discontinued treatment due to PD. As of the data cutoff date, 10 patients were still receiving anlotinib. All (100%) patients experienced at least one AE (**Table 3**). The most frequent AEs were hypertension (66.67%), hypothyroidism (54.76%), palmar-plantar erythrodysesthesia syndrome (40.48%), proteinuria (40.48%), and diarrhea (40.48%). Grade 3 and above AEs occurred in 23 (54.76%) patients, and included hypertension (19.05%), hypertriglyceridemia (9.52%) and pustulosis palmaris et plantaris (7.14%). In addition, SAEs occurred in 15 (35.71%) patients, of which 7 (16.67%) were treatment-related. Four (9.52%) deaths occurred, including 1 case each of brain metastasis, respiratory difficulty, pleural effusion and sudden death, all of which were not treatment-related.

**Table 3:** Adverse events (AEs) with an incidence ≥10% and grade 3 and above AEs.

Preferred Terms	All grades	Grade 3 and above
**Total**	42 (100.00)	23 (54.76)
**Hypertension**	28 (66.67)	8 (19.05)
**Hypothyroidism**	23 (54.76)	0 (0.00)
**Pustulosis palmaris et plantaris**	17 (40.48)	3 (7.14)
**Diarrhea**	17 (40.48)	0 (0.00)
**Proteinuria**	17 (40.48)	2 (4.76)
**Hypertriglyceridemia**	16 (38.10)	4 (9.52)
**Hypercholesterolemia**	12 (28.57)	0 (0.00)
**Elevated TSH**	11 (26.19)	0 (0.00)
**Reduced lymphocyte count**	10 (23.81)	1 (2.38)
**Fatigue**	9 (21.43)	0 (0.00)
**Hyperglycemia**	8 (19.05)	0 (0.00)
**Oropharyngeal pain**	8 (19.05)	0 (0.00)
**Reduced leucocyte count**	8 (19.05)	0 (0.00)
**Rash**	7 (16.67)	0 (0.00)
**Abdominal pain**	7 (16.67)	0 (0.00)
**Oral mucositis**	7 (16.67)	0 (0.00)
**Gingival bleeding**	7 (16.67)	0 (0.00)
**Reduced appetite**	7 (16.67)	0 (0.00)
**Elevated ALT**	7 (16.67)	0 (0.00)
**Elevated alkaline phosphatase**	6 (14.29)	1 (2.38)
**Elevated LDH**	6 (14.29)	0 (0.00)
**Reduced platelet counts**	6 (14.29)	2 (4.76)
**Reduced neutrophil counts**	5 (11.90)	1 (2.38)
**Reduced body weight**	5 (11.90)	0 (0.00)
**Elevated lipase**	(<10)	3 (7.14)
**Elevated amylase**	(<10)	2 (4.76)
**Elevated triglycerides**	(<10)	1 (2.38)
**Pneumothorax**	(<10)	1 (2.38)
**Oral ulcer**	(<10)	1 (2.38)
**Paraplegia**	(<10)	1 (2.38)
**Cancer pain**	(<10)	1 (2.38)

## Discussion

The current study demonstrated encouraging antitumor activities of anlotinib as second or later line of therapy for recurrent or metastatic primary malignant bone tumors. The 4-month PFS in patients with refractory or relapsed osteosarcoma patients with metastatic disease in the absence of targeted therapy has been reported to be 12% (3, 27, 28). In the current study, anlotinib achieved a 3-month PFS rate of 75.86% and a median PFS of 4.8 months in previously heavily treated advanced osteosarcoma patients, meeting the study primary endpoint. The PFS was comparable to that in the phase II trial for regorafenib (3.6 months, 95% CI, 2.0-7.6 months) and sorafenib (4 months, 95% CI 2-5 months) for advanced osteosarcoma (11, 13). The 3-month PFS rate of 75.86% achieved with anlotinib was also remarkable in comparison with the 4-month PFS rate of 46% achieved with sorafenib therapy (11). Despite notable improvement in PFS with anlotinib and, to a lesser extent, with regorafenib and sorafenib *versus* chemotherapy, the ORR remains disappointingly low with all three agents (6.89%, 14% and 13.6% for anlotinib, sorafenib and regorafenib, respectively), indicating limited tumor response with MKI monotherapy and highlighting the need for combination therapy such as with chemotherapy.

The DCR with anlotinib was 75.86% for advanced osteosarcoma in this trial, in contrast to 49% with sorafenib as reported earlier. All patients in the current study received adjuvant chemotherapy after surgery compared to 50% in the phase II trial on regorafenib. In a phase II study of refractory or metastatic soft tissue sarcoma, anlotinib achieved a median PFS of 5.6 months and an ORR of 13% (95% CI, 7.6–17) and a DCR of 74% (95% CI, 66–80) (22). Specifically, the median PFS was 5.6 and 11 months, respectively, for liposarcoma and leiomyosarcoma and the ORR was 7.7% in patients with both liposarcoma and leiomyosarcoma. In contrast, regorafenib achieved a median PFS of 1.1 and 3.7 months, respectively, for liposarcoma and leiomyosarcoma and no patients attained CR or PR in the REGOSARC trial, whereas sorafenib achieved a median PFS of 4.2 months for advanced soft tissue sarcoma (4.9 months for liposarcoma), with an ORR of 14.5% and a DCR of 47.4% (29, 30). Although different studies could not be compared directly, these findings suggested that anlotinib attained a numerically higher DCR with osteosarcoma and soft tissue sarcoma than regorafenib and sorafenib; however, randomized studies would be necessary to validate the superiority of anlotinib over other MKIs.

Though immune monotherapy has not demonstrated efficacy in osteosarcoma, the prospect of combining immune therapy and anti-angiogenesis MKI therapy appears promising. In a phase II trial in patients with advanced osteosarcoma progressing after chemotherapy, a combination of apatinib with camrelizumab, a humanized IgG4 κ monoclonal antibody against PD-1, achieved a median PFS of 6.2 months (95% CI 4.0-6.9 months), which is notably longer than 4 months with apatinib (31). Identifying patients with osteosarcoma who could benefit from immunotherapy with biomarkers such as PD-L1 or anti-angiogenesis MKI therapy could lead to further gains in patient survival.

Refractory or relapsed osteosarcoma patients fare very poorly due to lack of effective therapies (2). The unique bone microenvironment also renders immunotherapies largely ineffectual (32). Furthermore, osteosarcoma lacks targetable recurrent gene mutations and small molecules or therapeutic antibodies aiming at a single target may fail to deliver clinical benefits (6). The unique intrinsic properties of recurrent metastatic primary malignant bone tumors indicate that a multitarget inhibitor such as anlotinib or regorafenib, or a combinational approach with diverse mechanisms of actions is required for this patient population. In the current trial, anlotinib achieved a median PFS of 4.83 months (95%CI 3.48 to 7.13) for osteosarcoma. This compares favorably with other analogous agents (median PFS: apatinib, 4.5 months; lenvatinib, 3.4 months; regorafenib, 3.6 months) (11, 13, 33).

Recurrent or metastatic Ewing sarcoma has a very dismal outcome despite the best available therapeutic regimen including surgical resection and chemoradiotherapy (8). No (0/13) Ewing sarcoma in the SARC024 trial responded to regorafenib (13). In the current study, 2 out of the 3 patients with Ewing sarcoma/PNET achieved PR and 1 had SD, suggesting that anlotinib should be further explored as monotherapy or in combination with other agents for the treatment of Ewing sarcoma. The efficacy of anlotinib in Ewing sarcoma/PNET patients is comparable to that of anlotinib plus irinotecan for recurrent or refractory unresectable Ewing sarcoma (34). A small proportion of Ewing sarcoma patients are refractory to chemotherapy and anlotinib could offer an effective treatment for this subset of patients.

The incidence of grade 3 and above elevated triglycerides and hypertriglyceridemia was high (11.9%) in this trial. This is in line with previous studies of anlotinib for refractory solid tumors and refractory metastatic soft tissue sarcomas (22, 25). The rate of grade 3 and above hypertension (19.05%) in this trial is higher than that (4.2%) in a phase II study of 116 patients with refractory metastatic soft tissue sarcomas and that (10%) of phase I of 35 refractory solid tumors (22, 25). The rate of grade 3 and above pustulosis palmaris et plantaris (7.14%) is consistent with that of previous studies (22, 25). Notably, no treatment-related hematologic toxicities or bleeding events occurred. Grade 3 anemia (6%), leucopenia (3%) and bleeding (3%) were reported in osteosarcoma patients treated with sorafenib (11). Frequent hematologic toxicities were also reported for regorafenib, including grade 1 anemia (24%), lymphopenia (14%) and thrombocytopenia (10%). In addition, grade 3 hypophosphatemia and hypokalemia each occurred in 7% osteosarcoma patients treated with regorafenib. No new safety concerns emerged in this trial. Overall, the toxicity profile of anlotinib in this trial is consistent with the prior reports of anlotinib in other clinical studies and seems to be better than that of other oral anti-VEGFR TKIs (35).

This study has several limitations. First, this is a single arm study without a control group. In addition, the small sample size does not permit meaningful subgroup analyses to define the subset of patients who will truly benefit from anlotinib therapy. Furthermore, the study only included Han Chinese and the efficacy and safety of anlotinib in primary malignant bone tumor patients of other ethnicities remain yet to be defined.

In conclusion, anlotinib demonstrated promising antitumor activities and manageable safety profile in patients with recurrent or metastatic primary malignant bone tumors. The findings from this study support the use of anlotinib in patients with advanced primary malignant bone tumors who have progressed after chemotherapy, but further studies are needed.

## Data Availability Statement

The raw data supporting the conclusions of this article will be made available by the authors, without undue reservation.

## Ethics Statement

The studies involving human participants were reviewed and approved by The trial was approved by the institutional review board of Shanghai Sixth People’s Hospital (China). Written informed consent to participate in this study was provided by the participants’ legal guardian/next of kin.

## Author Contributions

Conceptualization: YY. Data curation: LT, XN, ZW, QC, CT, ZF and YY. Formal analysis: LT. Investigation: LT, XN, ZW, QC, CT, ZF and YY. Methodology: LT, YY. Project administration: YY. Resources: YY. Supervision: YY. Visualization: LT. Roles/writing - original draft: LT. Writing - review and editing: LT, XN, ZW, QC, CT, ZF and YY. All authors contributed to the article and approved the submitted version.

## Conflict of Interest

The authors declare that the research was conducted in the absence of any commercial or financial relationships that could be construed as a potential conflict of interest.

The reviewer QW declared a shared affiliation, with the author QC to the handling editor at time of review.

## Publisher’s Note

All claims expressed in this article are solely those of the authors and do not necessarily represent those of their affiliated organizations, or those of the publisher, the editors and the reviewers. Any product that may be evaluated in this article, or claim that may be made by its manufacturer, is not guaranteed or endorsed by the publisher.

## Appendix I List of Participating Centers

Shanghai 6th People’s Hospital, Shanghai 200233, China.

Beijing Jishuitan Hospital, Beijing 100035, China.

The First Affiliated Hospital, The Air Force Medical University, Xi’an 710032, Shaanxi Province, China.

Henan Cancer Hospital, Zhengzhou 450003, Henan Province, China.

West China Hospital, Sichuan University, Chengdu 610044, Sichuan Province, China.

Beijing Cancer Hospital, Beijing 100142, China. 
