# Supplementary material for: Anlotinib for Recurrent or Metastatic Primary Malignant Bone Tumor: A Multicenter, Single-Arm Trial
Source: Front Oncol. 2022 May 26;12:811687. doi: 10.3389/fonc.2022.811687 (PMC9177947; doi:10.3389/fonc.2022.811687)
Supplement: Supplementary file 1 [file DataSheet_1.docx]

**Supplementary Methods**

***Eligibility criteria***

Other inclusion criteria were 1) patients aged less than 18 years had a body surface area > 1.5 m^2^; 2) patients had a life expectancy > 3 months; 3) patients had adequate hematologic, cardiac, hepatic, and renal function within 7 days before the treatment: a) hemoglobin ≥ 90 g/L or ≥ 85 g/L in patients with anemia due multiple metastases; absolute neutrophil count ≥ 1.5×10^9^/L; platelet count ≥ 80 × 10^9^/L; b) total bilirubin≤ 1.5 × upper limit of normal (ULN); alanine aminotransferase (ALT) and aspartate aminotransferase (AST) ≤ 2.5×ULN (ALT and AST ≤ 5×ULN with liver metastasis); c) serum creatinine ≤ 1.5×ULN or creatinine clearance rate ≥ 60 mL/min; d) left ventricular ejection fraction (LVEF) ≥ 50% of lower limit of normal on Doppler echocardiography; 3) non-lactating women, and fertile women and men who agreed to use contraceptive measures during and within 6 months after the study.

Other key exclusion criteria were 1) pleural effusion/ascites ≥800 mL or causing respiratory difficulties (NCI CTC AE4.03 ≥ grade 2) concurrent malignancy or malignancy within the preceding 5 years, excluding cervical carcinoma *in situ*, skin cancer other than melanoma and superficial bladder carcinoma; 3) use within the preceding 4 weeks before study entry or planning to use systemic antitumor therapy during the study including cytotoxic therapy, signal transduction inhibitor therapy and immunotherapy (use of mitomycin C within 5 weeks of study entry); receipt of extended field radiotherapy within the preceding 4 weeks before study or limited field radiotherapy in the preceding 2 weeks before study; 4) any severe and/or uncontrolled diseases: a) pharmacologically uncontrollable hypertension (SBP≥150 mmHg, DBP≥100 mmHg); b) above NYHA class I myocardial ischemia or myocardial infarction, cardiac arrythmia (including QTc ≥480 ms) and ≥ class II congestive heart failure; c) active or uncontrolled severe infection (≥CTC AE grade 2); e) liver cirrhosis, decompensated liver disease, activate hepatitis or chronic hepatitis requiring antiviral therapy; d) renal failure requiring blood or peritoneal dialysis; f) a history of primary or acquired immunodeficiency, or a history organ transplantation; g) inadequately controlled diabetes (fasting plasma glucose > 10 mmol/L; h) urinary protein≥ 2+ and 24-hour urinary protein > 1.0 g; i) seizure requiring treatment; 5) receipt of major surgery, excisional biopsy or traumatic injuries within 4 weeks of study entry; 6) arterial/venous embolism within the preceding 6 months; 7) tumor thrombus; 8) the longest diameter of a single tumor ≥ 20 cm; 9) receipt of investigational anti-cancer therapy within 4 weeks of study entry; 10) other safety events or concomitant diseases that could interfere with the completion of the study judged by the investigator.

**Supplementary Table 1.** Dose modifications in the safety set.

| Patient No. | Cycle when anlotinib dose was modified | Reasons for dose modification | No. of cycles received | Drug-related |
| --- | --- | --- | --- | --- |
| 1 | 5 | Grade 3 pneumothorax | 7 | Yes |
| 2 | 5 | Grade 3 proteinuria | 8 | Yes |
| 3 | 7 | Grade 2 reduced platelet counts, grade 3 reduced lymphocyte count | 8 | Yes |
| 4 | 4 | Grade 3 hand-foot syndrome | 6 | Yes |
| 5 | 1 (Day 21) | Grade 3 reduced platelet counts | 8 | Yes |
| 6 | 7 | Grade 3 oral ulcer | 10 | Yes |
| 7 | 5 | Grade 3 hand-foot skin reaction | 8 | Yes |
